# The early diagnostic value of neutrophil to lymphocyte ratio and platelet to lymphocyte ratio in neonatal late-onset sepsis

**DOI:** 10.3389/fped.2025.1483522

**Published:** 2025-03-07

**Authors:** Qigai Yin, Jing Yin, Lu Shen, Qin Zhou, WeiDong Xu

**Affiliations:** ^1^ Department of Pediatrics, The People’s Hospital of Suzhou New District, Suzhou, Jiangsu, China; ^2^ Department of Pediatrics, The Affiliated Zhangjiagang Hospital of Soochow University, Suzhou, Jiangsu, China; ^3^ Department of Pediatrics, Lianyungang Clinical Medical College of Nanjing Medical University, Lianyungang, Jiangsu, China

**Keywords:** neonatal, diagnostic, LOS, NLR, PLR

## Abstract

**Purpose:**

The purpose of this study is to investigate the early diagnostic value of the neutrophil to lymphocyte ratio (NLR), platelet to lymphocyte ratio (PLR), and C-reactive protein (CRP) in neonatal late-onset sepsis (LOS), as well as to evaluate the combined diagnostic utility of these markers for the early detection of neonatal LOS.

**Methods:**

The late-onset sepsis of newborns admitted to the neonatal intensive care unit of our hospital were retrospectively collected. 142 children with Late-Onset Sepsis (LOS) were selected as the LOS group, 50 neonates with systemic infection were selected as the systemic infection group, 50 neonates who underwent physical examination were selected as the non-systemic infection group. The differences of NLR, PLR, platelet-to-neutrophil ratio (PNR), and C-reactive protein (CRP), Procalcitonin among the three groups were compared.

**Results:**

The levels of NLR and PLR in LOS group were significantly higher than those in systemic infection group and non-systemic infection group. The Receiver Operating Characteristic (ROC) curve result revealed that the area under ROC (AUC, Area Under Curve) of NLR for the diagnosis of LOS was 0.903. When the optimal cut-off value was 1.30, the sensitivity and specificity were 89.4% and 81.0%. The AUC of PLR for the diagnosis of LOS was 0.833. When the optimal truncation value was 57.86, the sensitivity and specificity were 92.3% and 68.0%. The AUC of CRP for the diagnosis of LOS was 0.876, and the sensitivity and specificity were 76.8% and 87.0% when the optimal cut-off value was 10.21 mg/dl. When NLR, PLR, and CRP were combined to diagnosis LOS, The AUC was 0.942, the sensitivity and specificity were 90.8% and 86.0%.

**Conclusions:**

The levels of NLR and PLR in the LOS were higher, which have certain value in the early diagnosis of LOS, and combined with CRP can improve the diagnostic efficiency.

## 1 Introduction

Neonatal sepsis (NS) is a systemic infection in newborns caused by pathogenic microorganisms entering the bloodstream through various routes (1, 2). When it occurs after 72 h of birth, it is classified as late-onset sepsis (LOS). NS is common in neonatal intensive care units (NICUs) and is a leading contributor to neonatal mortality. A 2018 epidemiological study across 12 countries reported an incidence of 2.2%, with a mortality rate ranging from 11% to 19% (3, 4). This highlights NS as a critical global public health concern.

Neonatal sepsis (NS) can affect multiple organ systems, including the digestive, respiratory, circulatory, and hematologic systems, often presenting with nonspecific symptoms. This complexity makes early identification based on clinical signs challenging. Moreover, neonates’ underdeveloped immune systems and immature organ structures contribute to their heightened susceptibility to infections. Without timely intervention, the infection can rapidly disseminate, progressing from asymptomatic to septic shock, disseminated intravascular coagulation, or even death. Therefore, early detection, accurate diagnosis, and prompt treatment are critical to reducing NS-related mortality. Although blood cultures are essential for detecting infection, sepsis is primarily a clinical diagnosis based on a life-threatening response to infection (5). Early detection of sepsis can be challenging, as blood cultures alone cannot confirm the presence of sepsis, and the diagnostic process relies on clinical criteria and biomarkers. Commonly used nonspecific markers, such as white blood cell count, offer suboptimal sensitivity and specificity. Additionally, emerging inflammatory markers like Interleukin 6 (IL-6), serum amyloid A, and CD64 face clinical limitations due to high costs and restricted detection conditions (6). Thus, the search for rapid, reliable, and specific biomarkers for NS diagnosis remains essential for improving clinical outcomes.

Recently, the NLR and PLR have been widely reported as reliable markers for various infectious diseases such as pneumonia and appendicitis. They have demonstrated value in the diagnosis, severity assessment, and prognosis of these diseases (7, 8). There have been reports indicating that NLR and PLR have good predictive roles in the diagnosis and assessment of adult sepsis (9). This suggests that NLR and PLR may also serve as predictive indicators for NS, providing a reference for early clinical diagnosis. In this study, we conducted a retrospective analysis of clinical data from 242 neonates, comparing the levels of NLR and PLR between those with LOS and those with systemic infections or non-infectious diseases. Moreover, these ratios were compared with the commonly used clinical marker CRP. The aim was to explore the potential application value of NLR and PLR in the early diagnosis of LOS.

## 2 Materials and methods

### 2.1 Sample collection

Retrospective data were collected from January 1, 2017, to December 31, 2020, at the Neonatal Intensive Care Unit of Lianyungang Hospital affiliated with Xuzhou Medical University. A total of 142 full-term neonates with LOS admitted during this period were selected as the LOS group. Additionally, 50 newborns with systemic infections admitted during the same period (presenting with signs of infection upon admission, with sepsis diagnosis excluded during hospitalization, including 38 cases of neonatal pneumonia and 12 cases of neonatal omphalitis) were chosen as the systemic infection group. Furthermore, 50 newborns undergoing outpatient examinations (either in neonatal outpatient clinics or pediatric health check-up clinics) were included as the non-systemic infection group.

#### 2.1.1 Inclusion criteria

(1) LOS Group: Neonates aged between 7 and 28 days, diagnosed according to the diagnostic criteria for neonatal LOS established by the Neonatology Group of the Chinese Pediatric Society in 2019. The diagnosis requires clinical manifestations and positive blood culture results. If the blood culture identifies pathogenic bacteria, it must meet the criteria of two consecutive cultures yielding the same pathogenic strain or a single positive culture with elevated inflammatory markers, along with antibiotic treatment for 5 days or more. The pathogenic bacteria include, but are not limited to, Coagulase-negative staphylococci (CoNS), Staphylococcus aureus, Escherichia coli, Klebsiella pneumoniae, and other commonly recognized neonatal pathogens.
(2) Systemic infection Group: Neonates aged between 7 and 28 days with signs of infection upon admission, with sepsis diagnosis excluded during hospitalization.
(3) Non-systemic infection Group: Neonates aged between 7 and 28 days undergoing outpatient examinations at our hospital’s neonatal outpatient clinics or pediatric health check-up clinics.
(4) Blood samples from all patients were collected on the day of admission, before the initiation of antimicrobial treatment, under aseptic conditions. Clinical data were complete.

#### 2.1.2 Exclusion criteria

(1) Gestational age less than 37 weeks, age less than 7 days, or greater than 28 days.
(2) Newborns with genetic metabolic disorders, chromosomal diseases, or congenital developmental abnormalities.
(3) Newborns with concomitant immune system disorders, hematologic disorders, or impaired liver or kidney function.
(4) Newborns who received antimicrobial or antiplatelet drug therapy before blood sampling.
(5) Newborns with a history of maternal transfusion during delivery or postnatal transfusion.
(6) Positive blood culture without clinical evidence of sepsis, considered as specimen contamination in newborns.

### 2.2 Sample information

We collected general clinical data for all newborns during hospitalization, including age, gestational age, gender, birth weight, delivery method, and Apgar scores at 1 and 5 min. Laboratory test results, including neutrophil count, lymphocyte count, platelet count, CRP, and PCT, were obtained for all three groups of newborns. The NLR, PLR, and PNR were calculated. All blood samples for testing were collected within 30 min of admission, prior to the initiation of antimicrobial treatment. Additionally, blood samples for blood culture, complete blood count, CRP, and PCT tests were collected simultaneously and sent for analysis.

### 2.3 Statistical analysis

Statistical analysis was performed using SPSS 24.0 software. Normally distributed quantitative data are expressed as mean ± standard deviation (±s), while non-normally distributed data are presented as median (interquartile range) [M(P25, P75)]. For normally distributed data with homogeneity of variance among the three groups, one-way ANOVA with LSD *post-hoc* tests was used for pairwise comparisons. For data without homogeneity of variance, Welch's ANOVA was applied, followed by Games-Howell *post-hoc* comparisons. Non-normally distributed data were analyzed using the Kruskal–Wallis test for overall comparison, with pairwise comparisons conducted via the Bonferroni method. Categorical data are presented as [number (%)] and compared using appropriate statistical tests. Multiple logistic regression was employed to identify independent risk factors for neonatal late-onset sepsis (LOS). The diagnostic value of each marker for neonatal LOS was assessed using receiver operating characteristic (ROC) curves. A significance level of *P* < 0.05 was considered statistically significant.

## 3 Results

### 3.1 Comparison of general clinical data among the three groups of newborns

In the comparative analysis of newborns, various factors including age, gestational age, birth weight, gender, delivery method, 1 min Apgar score, and 5 min Apgar score were scrutinized across three groups: the LOS group, the systemic infection group, and the non-systemic infection group. The examination yielded no statistically significant differences in these parameters (*P* > 0.05), as meticulously outlined in Table 1.

**Table 1:** Comparison of general clinical data among the three groups of newborns.

Indicator	LOS group (*n* = 142)	Systemic infection group (*n* = 50)	Non-systemic infection group (*n* = 50)	*χ^2^*	*P*
Age (Day)	10.89 ± 3.19	11.18 ± 2.16	10.34 ± 3.32	1.009	0.366
Gestational age (Week)	39.10 ± 1.11	39.39 ± 0.99	39.18 ± 1.04	1.301	0.274
Birth weight, (kg)	3.44 ± 0.44	3.52 ± 0.45	3.46 ± 0.57	0.568	0.567
Male [Number (%)]	84 (59.15)	30 (60.00)	26 (52.00)	0.896	0.639
Vaginal Delivery [Number (%)]	117 (82.39)	40 (80.00)	42 (84.00)	0.280	0.869
1 min Apgar score	9 (9,9)	9 (9,9)	9 (9,9)	3.549	0.170
5 min Apgar score	10 (10,10)	10 (10,10)	10 (10,10)	0.915	0.633
Oxygen saturation (%)	97.85 ± 1.65	98.02 ± 1.27	97.80 ± 1.50	0.304	0.738
Systolic blood pressure (mmHg)	75.32 ± 6.56	74.98 ± 5.47	76.82 ± 4.41	1.465	0.233
Diastolic blood pressure (mmHg)	46.65 ± 4.52	45.38 ± 4.64	46.10 ± 4.74	1.586	0.207
Heart rate (beats/min)	137.66 ± 10.37	139.58 ± 10.78	136.48 ± 8.01	1.237	0.292

### 3.2 The NLR, PLR, PNR, CRP, PCT among three groups

Examining the data presented in Table 2, the comparison among Neonatal LOS group, systemic infection group, and non-systemic infection group newborns revealed noteworthy trends. Specifically, NLR, PLR, PNR, CRP, and PCT exhibited higher values in the LOS group in comparison to both the systemic infection and non-systemic infection groups. Furthermore, these parameters were elevated in the systemic infection group compared to the non-systemic infection group, with these differences proving statistically significant (*P* < 0.05).

**Table 2:** Comparison of NLR, PLR, PNR, CRP, and PCT among three groups.

Indicator	LOS group (*n* = 142)	Systemic infection group (*n* = 50)	Non-systemic infection group (*n* = 50)	*F/H*	*P*
NLR	2.41 ± 0.95	1.14 ± 0.83	0.69 ± 0.43	95.545	<0.001
PLR	76.30 ± 17.55	58.39 ± 18.97	47.38 ± 18.03	54.676	<0.001
PNR	31.74 (26.23,40.08)	64.91 (37.26,99.32)	72.73 (53.16,103.85)	77.501	<0.001
CRP	12.55 (10.32,25.45)	9.63 (7.42,10.65)	0.27 (0.12,0.52)	133.709	<0.001
PCT	0.50 (0.18,1.13)	0.35 (0.16,0.60)	0.10 (0.68,0.15)	76.745	<0.001

NLR, neutrophil-to-lymphocyte ratio; PLR, platelet-to-lymphocyte ratio; CRP, C-reactive protein (mg/dl); PNR, platelet-to-neutrophil ratio; PCT, procalcitonin (ng/ml).

Pairwise comparisons further showed that PNR values were lower in the LOS group than in both the systemic infection and non-systemic infection groups, with these differences also statistically significant (*P* < 0.05). However, no significant difference was found between the systemic infection and non-systemic infection groups (*P* > 0.05), as shown in Table 3.

**Table 3:** Pairwise comparisons of NLR, PLR, PNR, CRP, and PCT among three groups.

Gropup	NLR	PLR	PNR	CRP	PCT
LOS group	2.41 ± 0.95	76.30 ± 17.55	31.74 (26.23,40.08)	12.55 (10.32,25.45)	0.50 (0.18,1.13)
Systemic infection group	1.14 ± 0.83	58.39 ± 18.97	64.91 (37.26,99.32)	9.63 (7.42,10.65)	0.35 (0.16,0.60)
*P*	<0.001	<0.001	<0.001	<0.001	0.131
LOS group	2.41 ± 0.95	76.30 ± 17.55	31.74 (26.23,40.08)	12.55 (10.32,25.45)	0.50 (0.18,1.13)
None infection group	0.69 ± 0.43	47.38 ± 18.03	72.73 (53.16,103.85)	0.27 (0.12,0.52)	0.10 (0.68,0.15)
*P*	<0.001	<0.001	<0.001	<0.001	<0.001
Systemic infection group	1.14 ± 0.83	58.39 ± 18.97	64.91 (37.26,99.32)	9.63 (7.42,10.65)	0.35 (0.16,0.60)
None infection group	0.69 ± 0.43	47.38 ± 18.03	72.73 (53.16,103.85)	0.27 (0.12,0.52)	0.10 (0.68,0.15)
*P*	0.008	0.002	0.363	<0.001	<0.001

NLR, neutrophil-to-lymphocyte ratio; PLR, platelet-to-lymphocyte ratio; CRP, C-reactive protein (mg/dl); PNR, platelet-to-neutrophil ratio; PCT, procalcitonin (ng/ml).

**Figure 1:**
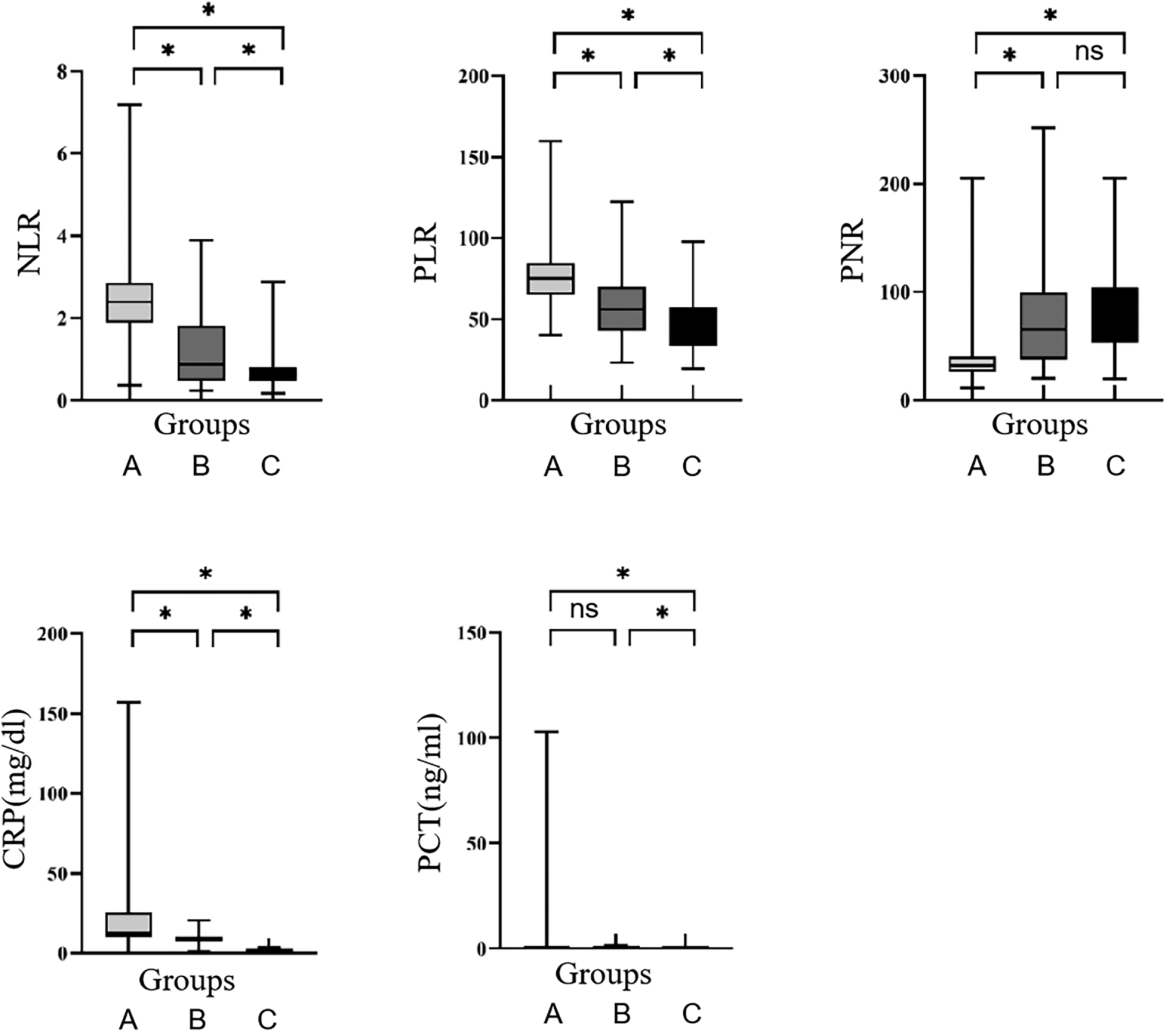
Comparison of NLR, PLR, PNR, CRP, and PCT among the three groups. **(A)** LOS group; **(B)** systemic infection group; **(C)** non-systemic infection group. *P < 0.05, ns, no significance.

Further analysis of PCT highlighted statistically significant variances between the LOS group and the non-systemic infection group, as well as between the systemic infection group and the non-systemic infection group (*P* < 0.05). Interestingly, no statistically significant difference was identified between the LOS group and the systemic infection group (*P* > 0.05), as depicted in Figure 1.

### 3.3 Logistic regression analysis

To ascertain independent factors influencing neonatal LOS, a multiple logistic regression analysis was conducted. The results revealed that NLR, PLR, and CRP were independent risk factors for the occurrence of neonatal LOS, while PNR and PCT were not independent influencing factors for neonatal LOS (Table 4). These results contribute valuable insights into the specific markers that independently contribute to the risk profile of neonatal LOS, enhancing our understanding of the underlying factors associated with this condition.

**Table 4:** Multiple logistic regression analysis of the independent factors influencing the occurrence of neonatal LOS.

Independent variable	*β*	Wald	OR value	95%*CI*	*P*
NLR	1.431	9.022	4.184	1.644–10.647	0.003
PLR	0.036	5.352	1.037	1.006–1.069	0.021
CRP	0.173	17.055	1.189	1.095–1.291	<0.001
PNR	0.003	0.173	1.003	0.988–1.020	0.678
PCT	0.605	1.363	1.832	0.663–5.057	0.243

NLR, neutrophil-to-lymphocyte ratio; PLR, platelet-to-lymphocyte ratio; CRP, C-reactive protein (mg/dl); PNR, platelet-to-neutrophil ratio; PCT, procalcitonin (ng/ml).

### 3.4 The diagnostic value of NLR, PLR, CRP, and combined indicators for neonatal LOS

**Figure 2:**
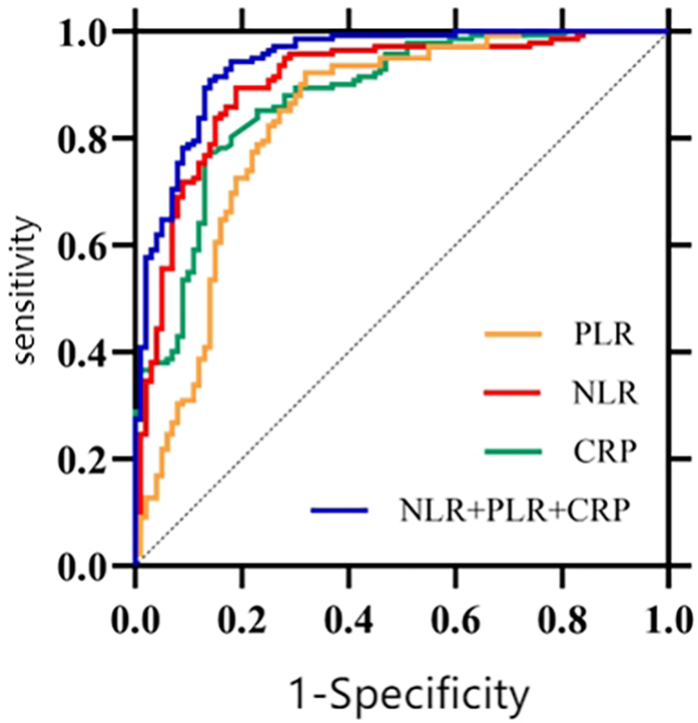
ROC curves for diagnosing neonatal LOS using NLR, PLR, CRP, and combined indicators.

Based on the results of the multiple regression analysis, Receiver Operating Characteristic (ROC) curves were constructed (Figure 2) to assess the predictive value of markers for neonatal late-onset sepsis (LOS). The Area Under the Curve (AUC) for NLR was 0.903, with an optimal cutoff of 1.30, achieving a sensitivity of 89.4% and specificity of 81.0%. For PLR, the AUC was 0.833, with an optimal cutoff of 57.86, yielding a sensitivity of 92.3% and specificity of 68.0%. CRP demonstrated an AUC of 0.876, with an optimal cutoff of 10.21 mg/L, resulting in a sensitivity of 76.8% and specificity of 87.0%. When combining NLR, PLR, and CRP, the AUC increased to 0.942, with sensitivity of 90.8% and specificity of 86.0%, as shown in Table 5.

**Table 5:** Diagnostic value of NLR, PLR, CRP, and combined indicators for neonatal LOS.

Indictor	AUC	Optimal cutoff	Sensitivity (%)	Specificity (%)	95% *CI*	*P*
NLR	0.903	1.30	89.4	81.0	0.862–0.944	<0.001
PLR	0.833	57.86	92.3	68.0	0.776–0.889	<0.001
CRP	0.876	10.21	76.8	87.0	0.831–0.920	<0.001
NLR + PLR + CRP	0.942	-	90.8	86.0	0.913–0.971	<0.001

NLR, neutrophil-to-lymphocyte ratio; PLR, platelet-to-lymphocyte ratio; CRP, C-reactive protein (mg/dl); PNR, platelet-to-neutrophil ratio; PCT, procalcitonin (ng/ml).

## 4 Discussion

Neonatal sepsis, particularly late-onset sepsis, continues to be a leading cause of morbidity and mortality in neonates, despite advances in neonatal care. Early diagnosis remains a critical challenge, and biomarkers such as the NLR and PLR have emerged as potential diagnostic tools. In this study, we focused on the diagnostic value of NLR and PLR in neonatal LOS, demonstrating their role as independent risk factors and providing new insights into their utility when combined with traditional inflammatory markers like CRP and PCT.

Elevated NLR has been identified as a significant marker in various forms of neonatal sepsis, with several studies pointing to its potential as an early diagnostic tool. Our study further supports this conclusion, finding that NLR was significantly higher in the LOS group compared to both systemic infection and non-systemic infection groups (*P* < 0.05). This reflects heightened neutrophil activity and lymphocyte depletion, both indicative of a robust inflammatory response in the context of neonatal sepsis. Our findings align with prior studies, such as those by C.D. Russell et al. in adult sepsis and E. Tamelytė et al. in pediatric sepsis, where elevated NLR was shown to correlate with disease severity and prognosis (10, 11).

A key contribution of our study is the specific focus on neonatal LOS, an area that has received less attention in the literature compared to early-onset sepsis (EOS). By examining the role of NLR in the diagnosis of LOS, we provide new evidence to support its diagnostic potential in this distinct population. Our results demonstrate that NLR is not only an early diagnostic marker but also an independent risk factor for neonatal LOS, as confirmed by multiple regression analysis. The ROC analysis, showing an AUC of 0.903 with sensitivity of 89.4% and specificity of 81.0%, further highlights NLR's strong diagnostic performance, comparable to findings in other studies on sepsis.

Interestingly, changes in NLR have also been reported in other co-infectious conditions, such as viral hepatitis. Xiao-Mao Li et al. have demonstrated that in patients with viral hepatitis, both NLR and PLR can also reflect the degree of inflammation and immune response (12, 13). In viral hepatitis, elevated NLR is often associated with more severe disease and worse prognosis, particularly in the setting of acute liver failure or chronic hepatitis. These findings underscore the broader relevance of NLR and PLR in various inflammatory and infectious diseases, further supporting their potential as universal markers of inflammation and immune dysfunction. However, the diagnostic thresholds and their specific roles in viral infections may differ from those in bacterial sepsis, as viral infections typically involve more complex immune responses, including altered lymphocyte subsets and cytokine profiles.

In addition to NLR, we explored the diagnostic utility of PLR in neonatal sepsis. Our study found that PLR was significantly elevated in the LOS group compared to the systemic infection and non-systemic infection groups (*P* < 0.05), suggesting its potential role as an inflammatory marker. PLR has been previously linked to disease severity in adult sepsis and has shown promise in predicting outcomes (14–17). In our study, logistic regression analysis identified PLR as an independent risk factor for neonatal LOS, with an AUC of 0.833, sensitivity of 92.3%, and specificity of 68.0%.However, while PLR showed promising diagnostic potential, our study also found that PNR did not serve as an independent risk factor for neonatal LOS, indicating its limited utility in this context. This suggests that, although platelet activation is central to inflammation and coagulation, PNR may not offer significant added value in the diagnosis of neonatal LOS, at least compared to NLR.

One of the major strengths of our study is the integrated approach of combining traditional inflammatory markers such as CRP and PCT with NLR and PLR. While CRP and PCT are widely used in clinical practice, they have limitations in early diagnostic accuracy, particularly for neonatal sepsis. Our study found that CRP was significantly elevated in the LOS group compared to the systemic infection and non-systemic infection groups (*P* < 0.05), confirming its diagnostic value as an independent risk factor for neonatal LOS (AUC = 0.876). PCT also showed elevated levels in the LOS and systemic infection groups compared to non-infected neonates (*P* < 0.05), though no significant difference was observed between the LOS and systemic infection groups.

Logistic regression further indicated that PCT was not an independent factor for neonatal LOS, highlighting the limitations of PCT as a sole diagnostic tool. However, when CRP was combined with NLR and PLR, the diagnostic efficacy was significantly enhanced, with an AUC of 0.942. This combined approach offers a novel contribution to the field, as it improves upon the diagnostic performance of individual markers. The integration of multiple biomarkers could provide a more robust tool for early and accurate detection of neonatal LOS, which is essential for improving clinical outcomes.

An important aspect of our study is the comparison of diagnostic criteria for neonatal sepsis between China and international standards. Our study followed the criteria established by the Neonatology Group of the Chinese Pediatric Society, which primarily relies on clinical manifestations and common inflammatory markers like CRP and PCT. However, international guidelines, such as the Phoenix Consensus, adopt a more comprehensive approach, incorporating multi-dimensional assessments including clinical scoring systems (e.g., pSOFA), organ dysfunction, and additional biomarkers like IL-6, IL-8, and PCT (5).

While our study demonstrated the significant diagnostic potential of NLR and PLR under the Chinese criteria, the broader, more dynamic diagnostic framework of the Phoenix Consensus may result in different performance for these markers. The international guidelines place greater emphasis on early organ dysfunction and inflammation beyond just neutrophil and platelet counts. This could potentially affect the relative weight and diagnostic thresholds of NLR and PLR in different clinical settings.

Despite the promising results of NLR and PLR, recent advancements in sepsis diagnostics have introduced a variety of novel biomarkers that could complement or even enhance these traditional indicators. New screening tools such as Presepsin (sCD14-ST), PTX3, nCD64, and Monocyte Distribution Width (MDW) have shown potential in improving sepsis detection (18–21). Among these, the LIP score, which incorporates biomarkers like lymphocyte count, INR, and PCT, is particularly noteworthy. Although the LIP score has shown promise in adult populations, its application to neonatal sepsis remains to be validated (22). The inclusion of these novel biomarkers alongside traditional ones could provide a more comprehensive diagnostic approach, offering both sensitivity and specificity in neonatal sepsis diagnosis (23). Future studies comparing the diagnostic performance of the LIP score and other emerging tools with the markers explored in our study would be beneficial in refining neonatal sepsis diagnostic protocols and improving clinical decision-making.

In conclusion, our study provides novel insights into the diagnostic value of NLR and PLR in neonatal LOS. These markers, when combined with traditional inflammatory markers like CRP and PCT, offer a promising approach to early and accurate diagnosis, which is critical for improving clinical outcomes. Our findings specifically address neonatal LOS, an area with less extensive research compared to early-onset sepsis. We believe that the integrated use of NLR, PLR, and traditional markers enhances diagnostic efficacy and may ultimately lead to more targeted interventions for neonates with suspected sepsis. As the field of sepsis diagnostics continues to evolve, future research will be essential to explore the role of emerging biomarkers and refine diagnostic protocols for neonatal sepsis.

## 5 Limitations

Despite the promising results, this study has several limitations. Firstly, it was a single-center, retrospective study, which may limit the generalizability of the findings to broader populations. Future multi-center, prospective studies with larger sample sizes are needed to validate these results and assess their applicability across different neonatal care settings. Secondly, while we focused on a range of biomarkers, there may be other inflammatory markers or biomarkers that could further enhance diagnostic accuracy but were not included in this study. Additionally, the lack of follow-up data on long-term outcomes in neonates with LOS means that we could not evaluate the prognostic value of these markers in predicting clinical outcomes such as survival rates or neurological development. Lastly, although NLR and PLR were identified as independent risk factors for LOS, the underlying biological mechanisms contributing to these changes were not fully explored, and further research into the pathophysiological role of these markers is warranted.

## 6 Conclusions

This study highlights the significant diagnostic value of the neutrophil-to-lymphocyte ratio (NLR) and platelet-to-lymphocyte ratio (PLR) in the early detection of neonatal late-onset sepsis (LOS). The ROC analysis revealed that NLR had an AUC of 0.903, with a sensitivity of 89.4% and specificity of 81.0%, while PLR demonstrated an AUC of 0.833, with a sensitivity of 92.3% and specificity of 68.0%. These results underscore the potential of NLR and PLR as reliable biomarkers for LOS diagnosis. Additionally, the combination of NLR, PLR, and CRP further improved diagnostic performance, yielding an AUC of 0.942. This combination may enhance clinical decision-making in identifying neonatal LOS at an early stage.

## Data Availability

The datasets presented in this study can be found in online repositories. The names of the repository/repositories and accession number(s) can be found in the article/Supplementary Material.
