# Supplementary material for: The early diagnostic value of neutrophil to lymphocyte ratio and platelet to lymphocyte ratio in neonatal late-onset sepsis
Source: Front Pediatr. 2025 Mar 7;13:1483522. doi: 10.3389/fped.2025.1483522 (PMC11925938; doi:10.3389/fped.2025.1483522)
Supplement: Supplementary file 1 [file Datasheet1.docx]

Statistics and analsis

Baseline data:

day age,

gestational age,

birth weight,

delivery mode,

etc

Laboratory indicators：

The neutrophil count,

lymphocyte count,

platelet count,

CRP and PCT

NLR

PLR

PNR

A total of 142 eligible neonates were enrolled

Exclusion Criteria

1) gestational age < 37 weeks, gestational age < 7 days or > 28 days;

(2) neonates with inherited metabolic diseases, chromosomal diseases or congenital malformations;

(3) neonates with immune system diseases, hematological diseases, liver and kidney dysfunction;

(4) neonates treated with anti-infective or anti-platelet drugs before blood collection;

(5) The mother had a history of blood transfusion during delivery or after birth;

(6) Newborns with positive blood culture but no clinical evidence of sepsis were considered as contaminated samples;

(7) incomplete clinical data;

The patient met the diagnostic criteria for neonatal LOS

Infants admitted to the neonatal intensive care unit (NICU) during the study period
